# Proximal Aortic Stiffness Is Increased in Systemic Lupus Erythematosus Activity in Children and Adolescents

**DOI:** 10.1155/2013/765253

**Published:** 2013-09-19

**Authors:** Yehia Mohamad El Gamal, Ola Abd Elaziz Elmasry, Iman Saleh El Hadidi, Ola Kamel Soliman

**Affiliations:** ^1^Pediatric Department, Faculty of Medicine, Ain Shams University, Cairo 11566, Egypt; ^2^Clinical Pathology Department, Faculty of Medicine, Ain Shams University, Cairo 11566, Egypt

## Abstract

Patients with systemic lupus erythematosus (SLE) are prone to premature atherosclerosis and are at risk for the development of cardiovascular disease. Increased arterial stiffness is emerging as a marker of subclinical atherosclerosis. *Purpose*. To measure proximal aortic stiffness in children and adolescents with SLE. *Methods*. We studied 16 patients with SLE in activity (mean age 15 ± 2.42 years; 16 females), 14 patients with SLE not in activity (mean age 15.7 ± 1.89 years; 4 males, 10 females), and 16 age- and sex-comparable healthy children and adolescents (15.5 ± 1.71 years; 4 males, 12 females). Disease activity was determined by the SLE disease activity index (SLEDAI). All subjects underwent echocardiography for assessment of proximal aortic pulse wave velocity (PWV) [Ao distance/Ao wave transit time in the aortic arch]. Venous blood samples were collected for ESR. *Results*. Patients in activity had significantly higher PWV values than controls (*P* < 0.05), while no significant difference was found between patients not in activity and controls. *Conclusions*. SLE patients with disease activity demonstrate increased PWV and arterial stiffness of the proximal aorta, while patients without disease activity do not. This suggests that inflammation secondary to SLE activity, and not subclinical atherosclerosis, is the major underlying cause for increased arterial stiffness in this age group.

## 1. Introduction

Patients with systemic lupus erythematosus (SLE) are prone to premature atherosclerosis, and the incidence of atherosclerosis-related myocardial infarction is as much as 50-fold greater in young patients with SLE than in age-matched controls [1]. Potential explanations for accelerated atherosclerosis in SLE include a high prevalence of conventional risk factors [2], long term corticosteroid use [3], the presence of antiphospholipid antibodies [4], and proatherogenic pathophysiologic phenomena inherent to SLE, such as dyslipidemia, immune system activation, endothelial cell apoptosis, oxidative stress [5], and chronic immune complex formation [6], all of which result in a chronic low grade inflammatory state, endothelial dysfunction, and, eventually, atherosclerotic events [5]. SLE patients should therefore be regarded as population at risk for the development of coronary artery disease, similar, for example, to patients with diabetes, in whom prompt identification and stringent treatment of risk factors are recommended [7, 8]. 

One of the recently accepted ultrasound-derived markers of early, asymptomatic atherosclerosis is increased arterial stiffness, as evidenced by increased pulse wave velocity (PWV) in the proximal aorta [9]. PWV may be considered an indirect measure of the biophysical properties of the aorta. The more compliant the central arteries are, the greater the percentage of stroke volume, that can be absorbed in systole to be released into the peripheral circuit in diastole is. The stiffer the artery is, the less it absorbs, and more of the stroke volume passes down the arterial system with a more rapid PWV [10].

Premature atherosclerosis has been previously described in children with SLE [11], and in the present study, we aimed to measure the proximal aortic stiffness and adolescents with SLE, its relationship to disease activity, and whether it can be used as an indicator of subclinical atherosclerosis in this patient population.

## 2. Patients and Methods

Patients included in this study were a consecutive cross-sectional sample, chosen from among children and adolescents with SLE following up at the Pediatric Immunology Clinic, the Children's Hospital. All patients and controls, as well as their parents, provided informed consent to participate in the study and the study protocol was ethically approved by the Pediatric Departmental Board.

The diagnosis of SLE was established using standard criteria [12] in 30 children (26 females; 4 males). We excluded patients with other forms of autoimmune disease or congenital heart disease. Sixteen clinically healthy age- and sex-matched children (12 females; 4 males) were also studied as a control group. 

A thorough clinical examination was performed for all children included in the study. Disease activity was determined by the systemic lupus erythematosus disease activity index (SLEDAI) [13], and patients were subsequently enrolled into either group A (in activity; *n* = 16) or group B (not in activity; *n* = 14). Venous blood samples were collected for full blood count and erythrocyte sedimentation rate.

**Figure 1:**
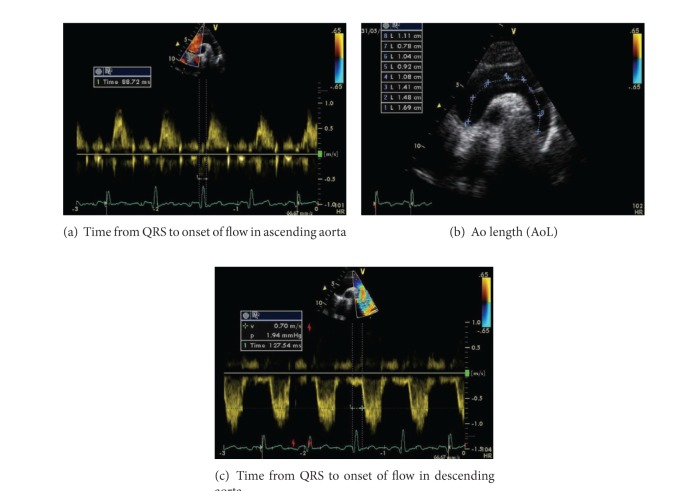
Calculation of PWV = AoL/TT (cm/sec), where AoL was the aortic arch length measured from the suprasternal arch (b) and TT (transit time) = (time from the beginning of the QRS to the onset of the PW Doppler envelope in the descending aorta)–(time from the beginning of the QRS to the onset of the PW Doppler envelope in the ascending aorta) = ((c)–(a)).

All patients and control subjects underwent echocardiography performed by an experienced pediatric echocardiographer (OAE) using GE Vivid 7 Dimension Echocardiography Machine (GE Medical System, N-3190, Horten, Norway). Complete two-dimensional (2D), motion-mode (M-mode), and Doppler echocardiographic examinations were performed according to published guidelines [14]. Pulse wave velocity (PWV) in the proximal aorta, reflecting proximal aortic stiffness [10], was measured using the following calculation: PWV = AoL/TT (cm/sec); where AoL was the aortic arch length measured from the suprasternal arch and TT (transit time) was time from the beginning of the QRS to the onset of the descending aorta PW Doppler envelope minus time from the beginning of the QRS to the onset of the ascending aorta PW Doppler envelope [15] (Figure 1).

### 2.1. Statistical Methods

Standard computer program SPSS for windows, release 13.0 (SPSS Inc., USA), was used for data entry and analysis. Comparison of different variables in various groups was done using Student *t*-test and Mann-Whitney *U* test for normal and nonparametric variables, respectively. Comparisons of multiple subgroups were done using ANOVA and Kruskall-Wallis tests for normal and nonparametric variables, respectively. Chi-square (*χ* ^2^) test was used to compare frequency of qualitative variables among the different groups. Spearman's correlation test was used for correlating nonparametric variables. For all tests probability (*P*) less than 0.05 was considered significant. 

## 3. Results

The descriptive data of the study groups are shown in Table 1 and the clinical manifestations both at initial presentation and at the time of our study in the patient groups are shown in Table 2. There was no significant difference among studied groups in any of the demographic features (*P* > 0.05). In group I (SLE in activity) 4 patients were in mild or moderate activity (SLEDAI < 11) and 12 patients were in high or very high activity (SLEDAI > 11).

All our patients were on glucocorticoid therapy (high dose in 13). Twenty-eight patients were on hydroxychloroquine therapy, 10 on cyclophosphamide, and 3 on azathioprine. Additionally, 28 patients were on regular angiotensin converting enzyme inhibitor therapy and 3 were on calcium channel blockers.

None of the patients or the controls had abnormal LV function. Eleven patients were found to have a pericardial effusion and 15 patients had valvular affection [isolated mitral regurgitation in 12, mitral regurgitation and mitral stenosis in 2, and aortic regurgitation in 1].

**Figure 2:**
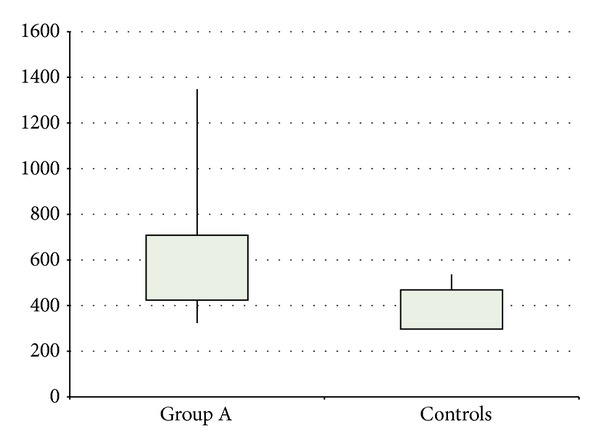
Comparison between patients in activity and controls as regards PWV. *P* < 0.05.

SLE patients in activity (group I) had significantly higher PWV values than controls (*z* − 2.13; *P* < 0.05) (Figure 2) while SLE patients not in activity did not (*z* − 1.15; *P* > 0.05). There were no significant correlations found between PWV and either SLEDAI or ESR in the patient groups.

## 4. Discussion

Accelerated atherosclerosis is considered an important cause of morbidity and mortality in patients with SLE [16] and highlights the importance of recognizing and controlling modifiable risk factors [17]. 

Pulsed wave velocity (PWV) of the proximal aorta is a relatively new technique for assessment of arterial stiffness [15]. We found that patients with SLE in activity had significantly higher PWV than controls. Sandor et al. [15] also reported elevated PWV in a group of children with inflammatory connective tissue disease (ICTD), including SLE. Similarly, in a study on older patients with SLE, PWV was also increased and factors associated with increased PWV were the severity of the disease in premenopausal women and age in postmenopausal women [18].

Although both studies implicate atherosclerosis as the cause of the increased PWV, the exact aetiology of this increased arterial stiffness in SLE is controversial [9, 19]. Although arterial stiffening may be an early event in the development of atherosclerosis, other mechanisms may be involved in the pathogenesis of arterial stiffening in SLE patients, in particular immunological mechanisms [9]. Prolonged periods of immune complex mediated manifestations, such as vasculitis and glomerulonephritis, associated with hypocomplementemia, render SLE patients more prone to develop arterial stiffness. Colburn et al. [20] reported diminished elastin production and enhanced elastin degradation in SLE. This would result in shifts in the collagen/elastin ratio in the vessel wall and increased arterial stiffness. Prospective longitudinal studies in SLE patients during activity and quiescence would help clarify whether the elevated PWV during activity is a marker of disease activity in this patient population or can be used as a very early marker of atherosclerosis. This mirrors the argument applied to conventional laboratory risk factors, such as C-reactive protein (CRP), which also fail to identify premature atherosclerosis in patients with SLE as they may be elevated due to the disease itself or infective complications [21]. Elevated PWV cannot therefore be used with certainty as an early marker of atherosclerosis in this young patient population; it probably reflects arterial inflammation which in itself can predispose to atherosclerosis.

Our study is limited by the small sample size and the absence of lipid profile assessments in our patients. 

In conclusion, SLE disease activity in children and adolescents is associated with increased PWV and arterial stiffness of the proximal aorta, while SLE without disease activity is not. This points to disease-induced inflammation as a cause for the increased arterial stiffness in children and adolescents with SLE disease activity, rather than early atherosclerosis. Further longitudinal studies are needed to determine whether increased PWV and arterial stiffness in SLE are reversible with resolution of disease activity, and whether/when increased PWV can be used as a marker of early atherosclerosis in this patient population. 

## Figures and Tables

**Table 1:** Descriptive data of studied groups.

Variable	Group I	Group II	Control group
	*n* = 16	*n* = 14	*n* = 16
Age (years)			
Mean ± SD (Range)	15 ± 2.42 (12–18)	15.7 ± 1.89 (13–18)	15.5 ± 1.71 (12–18)
Age at diagnosis of SLE (years)			
Mean ± SD (Range)	11.7 ± 1.7 (9–14)	11.6 ± 1.7 (2–15.2)	
Duration of SLE (years)			
Median (Range)	3 (0.5–9)	2.5 (0.2–7)	
Weight (kg)			
Mean ± SD (Range)	55.8 ± 12.37 (40–86)	55.9 ± 12.19 (38–84)	47.56 ± 8.6 (33–65)
Height (cm)			
Mean ± SD (Range)	141 ± 10.2 (128–160)	150.5 ± 11.13 (128–165)	149.8 ± 9.2 (130–160)
Male			
*n*	0	4	4
SBP (mmHg)			
Mean ± SD (Range)	139 ± 17.9 (110–170)	115 ± 14.9 (100–160)	111 ± 8.63 (90–127)
DBP (mmHg)			
Mean ± SD (Range)	88 ± 10.8 (65–100)	72 ± 7.26 (60–80)	67 ± 7.4 (56–80)
ESR (mm/hr)			
Median (Range)	95 (35–140)	19 (5–50)	14 (8–40)
SLEDAI			
Median (Range)	17.5 (2–71)		

Abbreviations: DBP: Diastolic blood pressure; ESR: erythrocyte sedimentation rate; SBP: systolic blood pressure; SLEDAI: systemic lupus erythematosis disease index.

**Table 2:** Clinical manifestations at initial presentation and at the time of the study in the patient group.

Manifestation	At initial presentation	At time of the study
	*n*	*n*
Skin/mucosal involvement		
Malar rash	20	
Discoid rash	1	
Oral ulcers	16	2
Photosensitivity	28	3
Musculoskeletal involvement		
Arthritis	28	11
Renal involvement		
Proteinuria	28	16
Serositis		
Pleuritis	5	3
Pericarditis	15	11
CNS involvement		
Seizures	3	3
Psychosis	4	5
